# The influence of behavioural and health problems on alcohol and drug use in late adolescence - a follow up study of 2 399 young Norwegians

**DOI:** 10.1186/1753-2000-5-17

**Published:** 2011-05-20

**Authors:** Arve Strandheim, Grete H Bratberg, Turid L Holmen, Lindsey Coombes, Niels Bentzen

**Affiliations:** 1 The Department of Public Health and General Practice, the Faculty of Medicine, Norwegian University of Science and Technology (NTNU), Trondheim, Norway; 2 School of Health and Social Care, Oxford Brookes University, Oxford, UK; 3 HUNT research centre, The Department of Public Health and General Practice, the Faculty of Medicine, Norwegian University of Science and Technology (NTNU), Levanger, Norway; 4 Department for Research and Development, Nord-Trøndelag Health Trust, Levanger, Norway; 5 Department of Child and Adolescent Psychiatry, Levanger Hospital, Nord-Trøndelag Health Trust, Levanger, Norway

## Abstract

**Background:**

Both early alcohol debut, behavioural and health problems are reported to enhance adolescence substance use. This prospective study investigate the influence of behavioural and health problems on adolescents' alcohol and drug use.

**Method:**

Prospective population based cohort study of 2 399 adolescents attending the Young-HUNT study, aged 13-15 at baseline in 1995/97, and 17-19 at follow-up 4 years later. Exposure variables were self reported conduct problems, attention problems, anxiety and depressive symptoms, and muscular pain and tension. Outcome variables at follow-up were frequent alcohol use and initiation of drug use. Associations were estimated by logistic regression models, influence of gender and drinking status at baseline were controlled for by stratification.

**Results:**

At follow-up 19% of the students drank alcohol once a week or more frequently. Baseline conduct problems (OR 2.2, CI 1.7-3.0) and attention problems (OR 1.5, CI 1.2-2.0) increased the risk for frequent alcohol use at follow-up in the total population. Girls who had experienced alcohol-intoxications at baseline showed strong association between baseline problems and frequent alcohol use at follow-up. Conduct problems (OR 2.5, CI 1.3-4.8), attention problems (OR 2.1, CI 1.2-3.4), anxiety/depressive symptoms (OR 1.9, CI 1.1-3.1) and muscular pain and tension (OR 1.7, CI 1.0-2.9) all were associated with frequent alcohol use among early intoxicated girls.

14% of the students had tried cannabis or other drugs at follow-up. Conduct problems at baseline increased the odds for drug use (OR 2.6, CI 1.9-3.6). Any alcohol intoxications at baseline, predicted both frequent alcohol use (boys OR 3.6, CI 2.4-5.2; girls OR 2.8, CI 1.9-4.1), and illegal drug use (boys OR 4.7; CI 3.2-7.0, girls OR 7.7, CI 5.2-11.5) within follow-up.

**Conclusions:**

Conduct problems in high-school more than doubles the risk for both frequent alcohol use and initiation of drug use later in adolescence. The combination of health problems and alcohol intoxication in early adolescence was closely associated with more frequent drinking later in adolescence among girls.

Overall, early alcohol intoxication was closely associated with both frequent alcohol use and drug use at follow up in both genders

## Introduction

European adolescents have increased their alcohol and drug intake during the last decades; in particular binge drinking and cannabis use has grown [1-3]. Alcohol and drug use in adolescence has been associated with several classes of health problems: externalizing disorders such as conduct problems and hyperactivity/attention problems [4-8]; internalizing disorders: depression, anxiety and suicidal behaviour; and physical complaints [2,9-13].

The discussion on casual connections between behaviour, health and substance use has traditionally been focused on alcohol and drug's negative effects on health [14,15]. That early alcohol début affects later health and addictive behaviour is well documented [16-19]. However some studies have suggested that pre-existing behavioural- and health problems facilitate the early initiation and later problematic use of alcohol and drugs [7,20-22]. Predictive factors for cannabis use and early drinking onset were described in two recent prospective studies, identifying conduct problems as important in both [23,24]. Other researchers emphasized the common background variables between substance use and health problems [25,26]. Reports supporting a more developmental perspective, were behaviour, health problems and substance use interacts at different ages during childhood and adolescence, have emerged the last decades [22,27,28]. Physical health problems have also been linked to substance use, particularly among females [29]. The pattern of female health disadvantage have been described and debated, but seem consistent in the adolescence population [30]. Research conducted exclusively with girls [31], has revealed a dose response relationship between physical symptoms and increasing alcohol and substance abuse.

The complex causal relationships between behaviour, health problems and substance initiation and use in adolescence, need to be addressed prospectively in a total population. Given the mentioned literature on health problems impact on alcohol- and drug use in adolescence, little is known about the mediating effects of gender differences and early alcohol intoxications.

This study aims to, in a prospective design, to study the effect of behavioural and health problems on late adolescence regular drinking and drug use. To explore the impact of gender and early drinking on the relationship between behavioural-, health problems and substance use, also were important aspects of the study.

## Methods

### Participants and study design

The county of Nord-Trøndelag situated in central Norway has about 127 000 inhabitants. From 1995-97, all students in junior high schools (13-16 years) and high schools (16-19 years) in the county were invited to participate in the Young-HUNT1 study, the youth part of the Nord-Trøndelag Health Study (HUNT) (ref http://www.ntnu.no/hunt), 9 131, 90% participated. Four years later, 2000-01 students in the last two years of high school or in vocational training, including the youngest students from Young-HUNT 1, were invited to Young-HUNT2. Of the 2 969 students eligible, 2 399 student*s *(81%) participated both in Young-HUNT 1 and Young-HUNT 2 and comprise the cohort of this study. The mean follow-up time was 3.9 years.

The comprehensive self-report questionnaire including questions on somatic and mental health and lifestyle factors was completed during a school hour both in Young-HUNT 1 and 2. The ethical committee only allowed questions concerning drug use for students in high school (16-19 years old).

**Figure 1:**
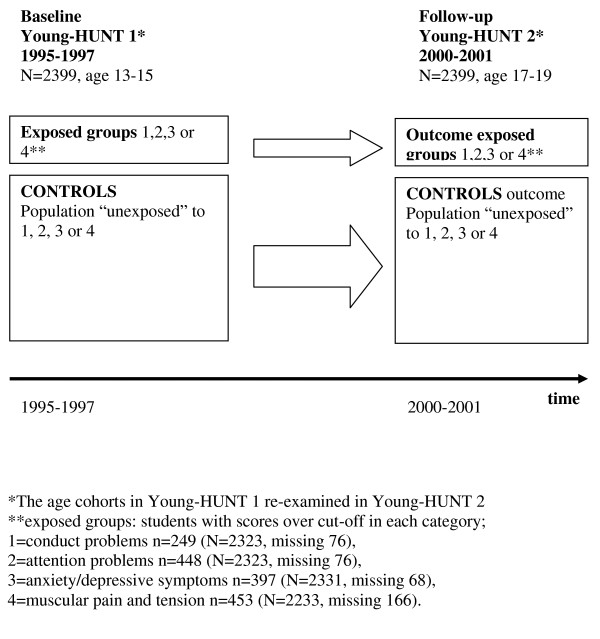
**Time line prospective cohort design Young-HUNT 1 & 2**. Exposed groups at baseline have either attention problems, conduct problems, anxiety and depressive symptoms or pain/tension problems over the 70^th ^percentile

A prospective cohort method was applied in the present paper using questionnaire data from the 2 399 students who participated both in Young-HUNT 1 (baseline) and in Young-HUNT 2 (follow-up). Data at baseline was used to create subgroups with high score on the different problem areas. Each subgroup was compared with the rest of the population without that problem behaviour, according to alcohol or drug use at follow-up (Figure 1).

### Measures

#### Baseline measures, exposure data (Young-HUNT 1, 1995/97)

The physical and mental health parts of the Young-HUNT 1 questionnaire were analyzed in cross-sectional studies [32,33], variables defined and their associations with alcohol intoxications described. The variables thus defined were used as to define baseline problem groups in this prospective study.

#### Behavioural and health related variables

This study used four health related variables: 1) attention problems, 2) conduct problems, 3) symptoms of anxiety and depression and 4) symptoms of muscular pain and tension. The variables were derived by factor analysis of parts of the Young-HUNT 1 questionnaire (described below). To define problem groups, scores above the 70th percentile were used, which is in accordance with similar studies [32,34]. Every group was defined from the total study population; some individuals are represented in more than one problem category (figure 1).

##### Anxiety and depressive symptoms

An abbreviation of the anxiety and depression part of the Symptom Check List 90-R, SCL-5 [34,35], was used to measure symptoms of anxiety and depression during the last 14 days. Based on a factor analysis with a limit of Eigenvalue at 1, the present study does not divide anxiety and depressive symptoms, but combines all five items ("Been constantly scared and uneasy", "Felt tense and restless" and "Worries too much about different matters"; "Felt hopeless when thinking of future" and "Felt down or sad") into a common anxiety/depression variable. All items had four alternative responses, ranging from one: not at all to four: extremely. The scores of all the five items were summarized and ranged from 5 to 20. These aggregated scores constituted no true interval scale, and therefore, in line with previous studies [34], the summarized scores were recalculated into dichotomous categorical variables. Sum scores above 8 were classified as high level of anxiety/depressive symptoms.

##### Attention- and conduct problems

Variables concerning attention- and conduct problems were derived from the school adjustment part of the questionnaire, including 14 items, described in a previous study [36]. The students were asked: "Do any of these (situations listed below) happen to you at school, or have it happened before?" with four alternative responses from one: never to four: very often. Factor analyses revealed two factors with eigenvalue >1. "Having trouble concentrating in class" and "Can not manage to be calm in class" indicating attention problems, and "Arguing with the teacher", "Having fistfights" and "Getting scolded by the teacher" indicating conduct problems. The summarized scores of all items in each category were dichotomised into low or high scores, defining "attentions problems" or "conduct problems" as having scores above the 70^th ^percentile of the Young-HUNT 1 population. According to this classification "attention problems" were present at a cut off point between four and five and "conduct problems" present between five and six.

##### Pain and tension symptoms

To measure pain and muscular tension the students were asked if they had any of the following problems during the last 12 months: headache, neck pain, muscle and joint pain or palpitations. All questions had four response categories, from one: "Never" to four: "Often". The values were summarized (range 1-16) and dichotomized, defining students with sum score above 9 as having high levels of pain and tension symptoms [37].

### Alcohol intoxications

Baseline alcohol experience was defined using number of lifetime alcohol intoxications before the age of 16. The students answered the question "Have you ever been drunk". The five response alternatives were: Never, Once, 2-3 times, 4-10 times, More than 10 times; "Early alcohol intoxication" was defined as having been drunk once or more.

### Follow-up measures, outcome data, Young-HUNT 2 (00/01)

#### Frequent alcohol use

At follow-up the students were asked about the frequency of their alcohol use ("How often do you drink alcohol?"), allowing five response categories (1. never, 2. less than once a month, 3. less than every second week, but more often than once a month, 4. every other week, 5. every week or more often). The outcome measure "frequent alcohol use" at follow up was defined as "drinking alcohol once a week or more".

#### Drug use

The question "have you ever tried hash, marijuana or related drugs" with alternatives yes or no was used as the outcome measure for drug use.

#### Statistics

The analyses presented are based on direct used or composite variables from the study questionnaire. Missing data were excluded from the analysis according to "completers only" principle. In our study variables 2.8%-6.4% of responses were missing (figure 1). No measures were repeated, thus binary logistic regression models were performed to correlate the behavioural and health problems at baseline with frequent alcohol use and initiation of drug use at follow-up. All analyses presented were carried out using SPSS 16.0.

Age was adjusted for in all analysis, only with a modest effect on the Odds Ratios (OR). All variables were first introduces in univariate logistic regression, than forced into the same model. Even if the behavioural and health variables not where interpreted as epidemiologically confounders, in a full multivariable model they all adjusted for each other.

Testing for interactions among the co-variates revealed some clinically important effect modifiers. Analyzing for frequent alcohol use, gender interacted with conduct problems and anxiety/depressive symptoms, alcohol intoxications at baseline interacted with conduct problems, attention problems, anxiety/depressive symptoms and pain/tension. Analyzing for drug use, all four variables interacted with baseline alcohol intoxication and anxiety/depressive symptoms interacted with gender. These problems were dealt with by dividing the genders, and stratifying the results by frequency of alcohol intoxication at baseline. The variables then were fitted separately in series of univariate models all corrected for age.

## Results

A total of 2 399 students completed the questionnaire in both waves of the study, 1 115 boys and 1 284 [38] girls. Anxiety and depressive symptoms, attention problems, pain and tension problems were more frequent among girls than boys. Only conduct problems were most frequent among boys (Table 1). At baseline totally 624 students (26%) reported having been intoxicated with alcohol.

**Table 1:** Distribution and prevalence of early alcohol intoxication and behavioural and health problems* divided by gender at baseline.

	Boys (N = 1115)			Girls (N = 1284)			
	
Exposure variables	N	%	95%CI	N	%	95%CI	p-value**
Anxiety/depressive symptoms	128	11.5	9.6-13.4	269	21.0	18.7-23.2	<0.0001
Attention problems	188	16.9	14.7-19.1	260	20.2	18.0-22.4	0.04
Conduct problems	181	16.2	14.0-18.4	68	5.3	4.0-6.5	<0.0001
Pain and tension problems	148	13.3	11.3-15.3	305	23.8	21.5-26.1	<0.0001
Early alcohol intoxication	267	23.9	21.4-26.4	357	27.8	25.4-30.3	0.044

*Problems with attention, conduct, pain/tension and anxiety/depressive symptoms

**p-value for gender differences, Pearson's Chi Square two-tailed

### Frequent alcohol use at follow up

At follow-up 24% among the boys and 15% among the girls, totally 459 students (19%), drank alcohol once a week or more often, which in this study was defined as frequent drinking.

Analyzing the total population by logistic regression, both attention problems (Odds Ratio (OR) 1.5, Confidence Interval (CI) 1.2 -2.0) and conduct problems (OR 2.2, CI 1.7-3.0) at baseline increased the likelihood for frequent alcohol use at follow-up.

Anxiety and depressive symptoms (OR 1.4, CI 1.0-2.0) together with pain and tension problems (OR 1.6, CI 1.1 -2.2) only increased the likelihood for frequent alcohol use slightly among girls (Table 2). Entering all variables in the same model, only left Conduct problems (OR 1.7, CI 1.3-2.4) and Early alcohol intoxication (OR 2.4, CI 1.9-3.1) significant. The explained variance (Nagelkerkes R^2 ^0.6) remained unchanged from the univariate analysis with only Early alcohol intoxication to the full model.

**Table 2:** Associations between early alcohol intoxication, behavioural- and health problems at baseline and the likelihood (age adjusted OR, 95% CI) of frequent alcohol use* at follow-up; stratified by gender.

	Total (N = 2399)			Boys (N = 1115)			Girls (N = 1284)		
	
Exposure variables	OR	CI	R^2**^	OR	CI	R^2^	OR	CI	R^2^
**Bivariate log.reg**									
Anxiety/depressive symptoms	1.1	0.8-1.4	0.002	1.0	0.7-1.6	0.003	1.4	1.0-2.0	0.006
Attention problems	**1.5**	1.2-2.0	0.01	1.1	0.9-1.3	0.01	1.1	0.9-1.4	0.02
Conduct problems	**2.2**	1.7-3.0	0.02	1.8	1.2-2,5	0.02	2.8	1.6-4.7	0.02
Pain and tension problems	1.3	1.0-1.7	0.007	1.3	0.9-2.0	0.007	1.6	1.1-2.2	0.01
Early alcohol intoxication	**2.7**	2.1-3.4	0.06	2.8	2.0-3.9	0.05	2.8	2.0-4.1	0.05
**Full model log.reg**									
Anxiety/depressive symptoms	0.9	0.7-1.0		1.0	0.6-1.6		1.0	0.6-1.5	
Attention problems	1.2	0.9-1.6		1.1	0.7-1.7		1.4	0.9-2.1	
Conduct problems	**1.7**	1.3-2.4		1.3	0.9-2.0		1.8	1.0-3.2	
Pain and tension problems	1.1	0.8-1.5		1.2	0.7-1.8		1.3	0.9-1.9	
Early alcohol intoxication	**2.4**	1.9-3.1		2.5	1.7-3.6		2.6	1.7-3.8	
			0.06			0.06			0.07

Bivariate analyzes first, all variables then entered in the same model.

*. Frequent use at follow-up, i.e. drinking alcohol at least once a week

**Nagelkerkes R Square

Adolescents who had been alcohol intoxicated when entering the study, drank more regularly at follow-up than those who had not (OR 2.7, CI 2.1-3.4). Gender interacted with conduct problems and anxiety/depressive symptoms (p = 0.011). Alcohol intoxications at baseline interacted with conduct problems, attention problems, anxiety/depressive symptoms and pain/tension (p = 0.001-0.006). Due to these clinically important interactions, participants were stratified according to gender and their drinking status at the entry of the study (Table 3). Girls in the early intoxication group accounted for the major part of the association of early behaviour and health problems with later regular alcohol use. Frequent alcohol drinking at follow-up was more common among girls who reported health or behavioural problems at baseline than those without such problems, given that they had been alcohol intoxicated early (conduct problems OR 2.5, CI 1.3-4.8, attention problems OR 2.1, CI 1.2-3.4, anxiety and depressive symptoms OR 1.9, CI 1.1-3.1, pain and tension problems OR 1.7, CI 1.0-2.9).

**Table 3:** Associations between behavioural- and health problems^2 ^at baseline and the likelihood (age adjusted OR, 95% CI) of frequent alcohol use¹ at follow-up; stratified by gender and alcohol use status at baseline^3^.

		*Boys*			*Girls*		
		
***Alcohol use status at baseline***^3^	Distress versus no distress^2^	OR	95% CI	P-value	OR	95% CI	P-value
	Anxiety/depressive	1.3	0.7-2.2	.39	0.9	0.5-1.6	.69
***No early alcohol intoxication***	Attention problems	1.2	0.7-2.0	.47	1.2	0.7-2.0	.57
	Conduct problems	**1.7**	**1.0-2.7**	**.038**	1.4	0.5-4.1	.55
	Tension problems	1.1	0.6-1.9	.82	1.2	0.6-2.1	.38
							
	Anxiety/depressive	0.7	0.3-1.5	.37	**1.9**	**1.1-3.1**	**.019**
***Early alcohol intoxications***	Attention problems	1.3	0.8-2.3	.32	**2.1**	**1.2-3.4**	**.005**
	Conduct problems	1.2	0.7-2.2	.43	**2.5**	**1.3-4.8**	**.008**
	Tension problems	1.3	0.7-2.4	.39	**1.7**	**1.0-2.9**	**.042**

¹Frequent use at follow-up, i.e. drinking alcohol at least once a week

^2^Behavioural- or health problems refers to baseline self reported problems, i.e. anxiety/depressive symptoms, attention problems, conduct problems and muscular pain/tension

^3 ^Had ever been alcohol intoxicated at the time point they entered the study

### Drug use at follow up

14% of the boys and 13% of the girls, totally 336 students (14%), had tried cannabis or other drugs at follow-up.

Analyzing the total study population by logistic regression, adolescents with conduct problems at baseline increased the odds for drug use at follow up (OR 2.6, CI 1.9-3.6) independent of gender. Specifically among boys, symptoms of anxiety/depression (OR 2.2, CI 1.4-2.5) and tension problems (OR 1.9, CI 1.2-2.3) increased the risk for later drug use (Table 4). Entering all variables in the same model, still Anxiety/depressive symptoms (OR 2.1, CI 1.3-3.6) and Conduct problems remains significant among boys, together with Early intoxication(OR 5.5 CI 4.1-7.4) for all the students. The Nagelkerkes R^2 ^only improves slightly from a model with only Early alcohol intoxication (R^2 ^0.16- 0.17).

**Table 4:** Associations between behavioural- and health problems* at baseline and the likelihood (age adjusted OR, 95% CI) for ever had tried hash, marihuana or other related drugs at follow-up; stratified by gender.

	Total (N = 2399)			Boys (N = 1115)			Girls (N = 1284)		
	
Exposure variables	OR	CI	**R**^2**^	OR	CI	**R**^**2**^	OR	CI	**R**^**2**^
**Bivariate log.reg**									
Anxiety/depressive symptoms	**1.6**	1.4-1.9	0.04	**2.2**	1.4-2.5	0.07	1.1	0.8-1.6	0.03
Attention problems	**1.7**	1.3-2.2	0.04	1.4	0.9-2.1	0.04	**2.0**	1.4-2.8	0.05
Conduct problems	**2.6**	1.9-3.6	0.06	**2.7**	1.8-2.2	0.09	**2.4**	1.4-4.3	0.04
Pain and tension problems	**1.5**	1.2-2.0	0.04	**1.9**	1.2-2.3	0.06	1.4	1.0-2.1	0.03
Early alcohol intoxication	**5.9**	4.5-7.8	0.16	**4.7**	3.2-7.0	0.15	**7.7**	5.2-11.5	0.18
**Full model log.reg**									
Anxiety/depressive symptoms	1.0	0.8-1.6		**2.1**	1.3-3.6		0.8	0.5-1.3	
Attention problems	1.1	0.8-1.5		0.7	0.4-1.2		1.4	0.9-2.2	
Conduct problems	**1.9**	1.4-2.8		**2.1**	1.3-3.3		1.4	0.7-2.7	
Pain and tension problems	1.1	0.8-1.5		1.1	0.6-1.8		1.1	0.7-1.7	
Early alcohol intoxication	**5.5**	4.1-7.4		**4.5**	2.-6.9		**7.3**	4.7-11.4	
			0.17			0.19			0.19

Bivariate analyzes first, then all variables entered in the same model.

* Behavioural- or health problems refers to baseline self reported problem.

**Nagelkerkes R Square

Adolescents with reported alcohol intoxications in secondary school more often tried out cannabis and other related drugs at high school, boys (OR 4.7, CI 3.2-7.0), girls (OR 7.7, CI 5.2-11.5).

All four health/behavior variables interacted with baseline alcohol intoxication (p = 0.001), enforcing a stratification based on presence of early alcohol intoxication or not (Table 5).

**Table 5:** Associations between behavioural- and health problems^2 ^at baseline and the likelihood (age adjusted OR, 95% CI) for ever had tried hash, marihuana or other related drugs at follow-up; stratified by alcohol use status at baseline^3^.

*Alcohol use status at baseline*^3^	distress^2 ^versus no distress (ref.)	OR	95% CI	P-value
	Anxiety/depressive	1.2	0.8-2.0	0.4
***No early alcohol intoxication***	Attention problems	1.3	0.9-2.1	0.2
	Conduct problems	**2.7**	**1.6 -4.4**	**0.001**
	Tension problems	1.3	0.8-2.0	0.3
				
	Anxiety/depressive	1.2	0.8-1.8	0.3
***Early alcohol intoxications***	Attention problems	1.2	0.9-1.8	0.2
	Conduct problems	**1.6**	**1.0-2.4**	**0.04**
	Tension problems	1.2	0.8-1.7	0.4

¹Frequent drinking at follow-up, i.e. drinking alcohol at least once a week

^2^Behavioural- or health problems refers to baseline self reported problems, i.e. anxiety/depressive symptoms, attention problems, conduct problems and muscular pain/tension

^3 ^Had ever been alcohol intoxicated at the time point they entered the study

Conduct problems among adolescence not intoxicated at baseline nearly increases the odds for drug use three-fold. Anxiety/depressive symptoms also interacted with gender (p = 0.01).Due to the interaction and to compare with table 3 using frequent alcohol as outcome, stratification on both gender and baseline intoxication was performed (Table 6).

**Table 6:** Associations between behavioural- and health problems^2 ^at baseline and the likelihood (age adjusted OR, 95% CI) for ever had tried hash, marihuana or other related drugs at follow-up; stratified by gender and alcohol use status at baseline^3^.

		*Boys*			*Girls*		
		
*Alcohol use status at baseline*^3^	distress^2 ^versus no distress (ref.)	OR	95% CI	P-value	OR	95% CI	P-value
	Anxiety/depressive	**2.4**	**1.2-4.5**	**.009**	0.7	0.3-1.5	.38
***No early alcohol intoxication***	Attention problems	**2.0**	**1.1-4.0**	**.018**	0.8	0.4-1.7	.58
	Conduct problems	**2.7**	**1.5-4.9**	**.001**	1.8	0.5-6.2	.35
	Tension problems	1.3	0.6-2.7	.51	1.4	0.7-2.7	.29
							
	Anxiety/depressive	2.0	1.0-4.0	.06	1.0	0.6-1.7	.93
***Early alcohol intoxications***	Attention problems	0.6	0.3-1.2	.15	**1.9**	**1.2-3.1**	**.007**
	Conduct problems	**1.8**	**1.0-3.1**	**.050**	1.3	0.7-2.6	.45
	Tension problems	1.6	0.9-3.0	.14	1.0	0.6-1.7	.96

¹Frequent drinking at follow-up, i.e. drinking alcohol at least once a week

^2^Behavioural- or health problems refers to baseline self reported problems, i.e. anxiety/depressive symptoms, attention problems, conduct problems and muscular pain/tension

^3 ^Had ever been alcohol intoxicated at the time point they entered the study

Among boys not yet alcohol intoxicated at baseline, also anxiety/depressive symptoms increased the risk for initiation of drug use, compared to with boys with few problems (Table 4).

### Smoking and parental education

Smoking at baseline was associated with both frequent drinking (OR 2.3, CI 1.6-3.2) and drug use (OR 3.0, CI 2.1-4.3) at follow up. Correction for smoking did only modestly reduce the other relationships shown. There was no statistical relation between parental education and frequent drinking in high school (OR 1.1, CI 0.9-1.4), and only a modest relationship with drug use (OR 1.3, CI 1.0-1.7) estimated by logistic regression (age and gender corrected).

## Discussion

### Summary of main findings

Both health-related problems and alcohol intoxications in early adolescence showed influence on frequent alcohol use and initiation of illegal drugs 4 years later, with important gender differences. Any experience with alcohol intoxication in high school was strongly associated with both later frequent alcohol use and initiation of drug use, as expected [17,19]. Conduct problems in early adolescence also appeared to be a major contributor to increased risk for both frequent drinking and drug use in accordance with recent findings [8,24].

Girls' drinking in late adolescence was strongly affected by their reported health problems, only if they have experienced early alcohol intoxications. This effect was not demonstrated among girls without alcohol intoxications before the age of 16. Boy's drinking in late adolescence was mainly influenced by early alcohol intoxications and to some extent conduct problems in early adolescence. Boys showed associations between reported affective problems and drug use 4 years later, if they where unexposed to alcohol intoxication at baseline. This might appear to be somewhat in contradiction to earlier findings [26], but can be viewed as an indicator of the strength in the association of early alcohol involvement and later drug use. Early alcohol involvement is a well known, and such a potent precursor of later substance use [19], that only the group without early alcohol experience can reveal weaker causal relation among boys. Longitudinal studies support the view that with an earlier baseline, the effects of mental health factors could be detectable in the whole population [7]. Boy's relationship between affective problems and drug use might also be interpreted as self- medication, as discussed in conflicting earlier reports [39,40].

Adolescence alcohol and drug use seemed woven into health and behavioural problems, possibly both as consequences and casual factors

### Strengths and Limitations

The Young-HUNT Study is a prospective cohort study of a total teenage population with a high response rate. The most important strength of the study was the prospective design, covering an important period of adolescence where most health related lifestyle habits were established. The 4 years between the age of 14 and the age of 18 represents huge changes and possibilities for preventive strategies.

Overall early smoking and alcohol drinking is a known and dominating predictive factors for later alcohol and drug use. To reveal other important causative factors or possible synergetic effects, stratification in groups with or without early drinking experience was used. In the fully stratified models N in each cell was low; power is reduced and even statistically significant differences must be interpreted with caution.

The study has a possible socioeconomic bias capturing a higher percentage of the students than adolescents in vocational training. To explore this, an additional analysis correcting for family socioeconomics, using parental highest education was conducted. The statistical relationship between parental education and frequent drinking as well as drug use, was limited and did not alter the main findings in the article.

## Conclusions

This study supports the opinion that especially conduct problems, but also to some extent attention problems, anxiety/depressive symptoms and bodily pain in early adolescence might increase the risk for later substance use. Early alcohol experiences synergetic with health related problems influences drug and alcohol habits on the step to adulthood. Alcohol intoxication in early adolescence seems to activate vulnerability in girls with co-existing health problems. Boys with anxiety or depressive problems demonstrated higher risk for initiation of drug habits.

In accordance with previous findings [19,23,41] our study confirms that early alcohol intoxications or binge drinking substantially increases the odds for frequent alcohol and drug use later in adolescence. In that way our study might support the generally accepted goal in universal prevention programs; to reduce alcohol accessibility and postpone alcohol debut in the adolescent population. This might reduce the lifelong drug-related risk for the whole population.

Recent development of targeted preventive interventions addressing either the total adolescent population or indicated groups, have shown promising and lasting effects both on behavioural problems, alcohol and drug use (e.g. Strengthening Families Program10-14)[42,43]. Gender differences in the development of adolescence substance use visualize the need for further research and might require specific prevention-programs. Substance use initiation in adolescence appears so closely linked to other behavioural and health problem, that division in different compartments of health and social services seems groundless. With respect to future years of suffering and the costs of health services, further investigation in the interface between adolescence drug use and health is urgently needed.

## Competing interests

The authors declare that they have no competing interests.

## Authors' contributions

AS: development of idea and design, literature search, statistical analysis and writing the drafts for the manuscript. GB: idea development, statistics and presentation of the findings, TLH: PI of the young-HUNT study, development of the idea and supervision of method, LC: idea development, analysis and presentation, NB: development of idea, presentation, main supervisor.

All authors have contributed to the writing of the manuscript, and have approved the final version.
